# Reduced Haemodynamic Response in the Ageing Visual Cortex Measured by Absolute fNIRS

**DOI:** 10.1371/journal.pone.0125012

**Published:** 2015-04-24

**Authors:** Laura McKernan Ward, Ross Thomas Aitchison, Melisa Tawse, Anita Jane Simmers, Uma Shahani

**Affiliations:** Department of Vision Sciences, Glasgow Caledonian University, Glasgow, United Kingdom; Medical Photonics Research Center. Hamamatsu University School of Medicine, JAPAN

## Abstract

The effect of healthy ageing on visual cortical activation is still to be fully explored. This study aimed to elucidate whether the haemodynamic response (HDR) of the visual cortex altered as a result of ageing. Visually normal (healthy) participants were presented with a simple visual stimulus (reversing checkerboard). Full optometric screening was implemented to identify two age groups: younger adults (n = 12, mean age 21) and older adults (n = 13, mean age 71). Frequency-domain Multi-distance (FD-MD) functional Near-Infrared Spectroscopy (fNIRS) was used to measure absolute changes in oxygenated [HbO] and deoxygenated [HbR] haemoglobin concentrations in the occipital cortices. Utilising a slow event-related design, subjects viewed a full field reversing checkerboard with contrast and check size manipulations (15 and 30 minutes of arc, 50% and 100% contrast). Both groups showed the characteristic response of increased [HbO] and decreased [HbR] during stimulus presentation. However, older adults produced a more varied HDR and often had comparable levels of [HbO] and [HbR] during both stimulus presentation and baseline resting state. Younger adults had significantly greater concentrations of both [HbO] and [HbR] in every investigation regardless of the type of stimulus displayed (p<0.05). The average variance associated with this age-related effect for [HbO] was 88% and [HbR] 91%. Passive viewing of a visual stimulus, without any cognitive input, showed a marked age-related decline in the cortical HDR. Moreover, regardless of stimulus parameters such as check size, the HDR was characterised by age. In concurrence with present neuroimaging literature, we conclude that the visual HDR decreases as healthy ageing proceeds.

## Introduction

Ageing research is a significant area of scientific focus, yet there is a lack of clarity regarding the basic haemodynamic response (HDR) of the visual cortex as a function of healthy ageing. It is fundamental to understand low-level visual processes, which subsequently influence higher processing and perception. When examining simple visual stimulation, perhaps it can be expected that healthy optically corrected eyes will provide a standard visual cortical response. However, there is a substantial body of functional Magnetic Resonance Imaging (fMRI) work that suggests an age-related attenuation of the HDR from the primary visual cortex (V1). In old adults (over 65 years old) there is a reduction in V1 activation using either reversing checkerboards as stimuli[1–4] or a flashing light[5–7]. An age-related decline in V1 amplitude of HDR, increased variability in peak amplitude and response latency, reduced spatial activation, and increased signal-to-noise has been shown by various authors[2,4,7]. More recently, Ances et al., (2009) reported old adults to have a lower blood oxygenated level dependent (BOLD) signal compared to young adults, yet subjects were comparable regarding other physiological measures such as cerebral blood flow (CBF) and cerebral metabolic rate of oxygen consumption[8]. Their ‘old’ sample was ‘young’, with a mean age of 53. Therefore, caution is required for the interpretation of MRI findings reported exclusively in terms of the magnitude of the BOLD response, in addition to varying definitions of old and young sample ages. Yet, there is some neuroimaging evidence that indicates an age-related reduction in resting CBF. Using Transcranial Doppler ultrasonography (TCD) measuring the middle cerebral artery, the Rotterdam Study of 1, 720 adults aged between 65 and 90 reported a lower resting CBF in older adults[9]. The sample was split into 5-year age groups (e.g. 75–80) and even within each subgroup, CBF values declined significantly per year. Similarly, using continuous arterial spin labelling MRI, Bertsch et al., (2009) report a reduced resting CBF in older participants (mean age 64) in comparison to their younger counterparts (mean age 25)[10]. As such, there is literature using various neuroimaging techniques that shows a reduced resting state in brain activity amongst older adults.

Functional Near-Infrared Spectroscopy (fNIRS) is a non-invasive optical neuroimaging technique that captures the slow HDR associated with neural firing. fNIRS measures continuous changes of concentrations of chromophores ([chromophore]) in the blood: oxygenated [HbO], deoxygenated [HbR] and total haemoglobin [HbT], a sum of the previous two, providing excellent temporal resolution but poor spatial resolution. In brief, fNIRS relies on the differential absorption of near-infrared light by haemoglobin. Changes in scattering and absorption of light are calculated and [HbO] and [HbR] are determined (for more detail see[11,12]). This technique offers several advantages over fMRI as a system to study ageing: reduced sensitivity to motion artefacts, increased patient tolerance to environment, increased patient inclusion and lastly the power to separate [HbO] and [HbR]. Visual cortex activity was first measured by NIRS in response to light stimulation[11,13], wherein during stimulation subjects displayed a characteristic increase of [HbO] and decrease of [HbR] in the occipital surface. This distinctive response is seen with reversing checkerboard stimulation, which produces the strongest cortical activity compared to other visual stimuli[14–18]. However, using fNIRS there is mixed evidence regarding the specific effect of ageing on V1. Using NIRS to record over V1, Schroeter et al., (2004) found that regardless of age, all subjects showed a characteristic HDR: during visual stimulation there was an increase of [HbO] and decrease in [HbR][19]. However, using the same stimulus, sample age and a multichannel NIRS system, Fabiani et al., (2014) reported an age-related decline in magnitude of V1 response. This study employed multiple neuroimaging modalities and concluded age to be related to reduced neurovascular coupling. fNIRS data showed distinct age differences with older adults presenting with a reduced HDR compared to their younger counterparts[20].

When investigating the visual system it is important to consider both optical and neurological properties. As such, we specifically recruited healthy individuals and recorded measures such as visual acuity (VA) and contrast sensitivity function (CSF) to fully encapsulate each individual’s visual function. One of the most effective methods of measuring a person’s vision is by finding the boundary between images that can be seen and those that cannot. This limit, or threshold, is often defined as the smallest letter on an eye chart that can be named, where it is known as acuity. A more detailed picture of person’s vision can be obtained if we find the faintest pattern that they can see. The spatial CSF, which describes how grating sensitivity (1/threshold) varies with spatial frequency, is fundamental to basic and clinical vision science. Contrast sensitivity deficits accompany many visual neuro-pathologies, even when acuity or perimetry tests appear normal. Contrast sensitivity is also an important outcome measure for refractive and cataract surgery and potential visual rehabilitation programs. In the current study, to further elucidate the impact of healthy ageing on the visual HDR, we report the effect of varying basic visual stimulus characteristics (contrast and spatial frequency) while recording absolute values of [HbO] and [HbR] using fNIRS. Our aim was to see differences in visual stimulus characteristics that would reflect physiological activity of V1 rather than a reduced perception of high spatial frequency content, which is associated with a decline in age related visual acuity[21]. We hypothesized that there would be a difference in HDRs between age groups, and specifically that older adults would present with a reduced cortical response.

## Methods

### 2.1. Participants

We recruited 25 healthy participants: 12 young adults (mean age 20.5 ± 3 years, range 18–27, 12 females) and 13 old adults (mean age 71.2 ± 7 years, range 58–83; 8 females) from the Eye Clinic, Glasgow Caledonian University. All participants had visual acuity (VA) of at least 6/9 with optical correction when required, and were free from any history of neurological or psychological disorder. The average visual acuity for the young group was 6/4.8 and for the older adults 6/6. Past medical history was taken and medication noted, 6 of the 13 older adults were on hypertensive medication for high blood pressure, however this has previously been shown not to effect the visual HDR[22]. Additionally, no participants were anaemic, leading the authors to believe [chromophore] recorded from each individual to be an accurate measurement. The research protocol was approved by Glasgow Caledonian University’s Ethics Committee, and informed written consent was obtained from all participants prior to testing in accordance with the Declaration of Helsinki.

### 2.2. Procedure

#### 2.2.1 Visual Assessment

The older adults underwent a full optical assessment by a trained optometrist and were considered healthy without any significant ocular pathology. Visual field examinations were performed using Humphreys Field Analyser (HFA II SITA), a fast perimetry assessment of the central 25°. Visual acuity was assessed using a Snellen Chart and corrected acuity recorded, these were subsequently converted to logMAR values, e.g. 6/6 becomes 0.00. In addition to standard optometric assessments, all subjects completed psychometric testing to determine their contrast sensitivity function (CSF) using a quick methodology[23]. The principle underlying this paradigm is the use of a Bayesian adaptive algorithm to identify optimal spatial frequency and contrast levels ahead of each trial, chosen from a predefined space of finely sampled stimulus intensities. Starting from a typical contrast sensitivity function, based on VA testing data and population norms, the paradigm facilitates rapid estimation of the observer’s CSF. Both spatial frequency and contrast were varied with log-linear spacing (0.33–16 cycles per degree, cpd, for spatial frequency, 16 possible choices; 0.10–100% contrast, 60 possible choices) on each trial, subject to whether the previous response was correct or incorrect. By estimating the CSF parameters, responses to a single grating were recorded (participants had to indicate whether a grating was contained in the first or second interval). From this data a CSF curve was produced based on threshold contrast at each spatial frequency, and the area under this curve calculated as the AULCSF (area under line CSF). Additionally, this test was assessed under similar conditions as the fNIRS task in a dimly lit room (scotopic conditions) providing a comparable estimation of different aspects of the visual system.

#### 2.2.2. Instrumentation

Changes in [HbO] and [HbR] were measured using a two-channel, frequency-domain multi-distance (FD-MD) tissue oximeter (OxiplexTS^TM^). This fNIRS system measures the change in the intensity modulation and phase shift as a function of the distance of the emitting light source, scattering and absorption of the near infrared light. The OxiplexTS has 2 detectors and 16 emitters at different distances (8 at 690nm 8 at 839nm) and operates at an intensity modulation frequency of 110 MHz. Calibration is performed prior to testing on an optical phantom of known optical properties similar to those of brain tissue. Absorption and scattering coefficients are calculated for each wavelength and [chromophore] determined using the modified Beer-Lambert Law. The principles behind this system and the FD-MD approach have been described in detail elsewhere[12,14,24]. There is evidence that the FD-MD fNIRS system has the ability to separate haemoglobin concentrations in brain tissue and those from scalp and skull[25]. Another distinct advantage of this FD-MD fNIRS is that recordings are absolute measures of [chromophores], which are not jeopardized by assumptions such as differential path-length factor used in other NIRS systems, making it a particularly useful tool to study ageing.

#### 2.2.3. HDR Recording

**Fig 1:**
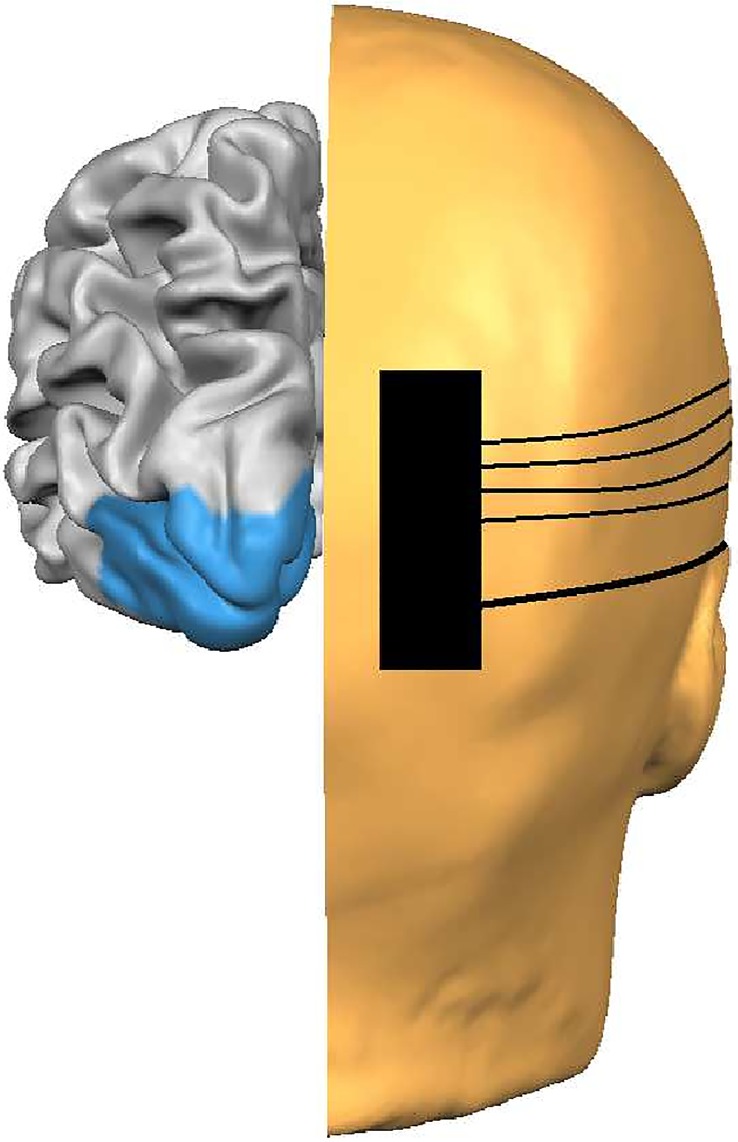
Diagram of fNIRS set-up with the sensor placed over O2 (right hemisphere, 10–20 EEG system). All recordings were taken from over both right (O2) and left (O1) primary visual cortices (blue region).

**Fig 2:**
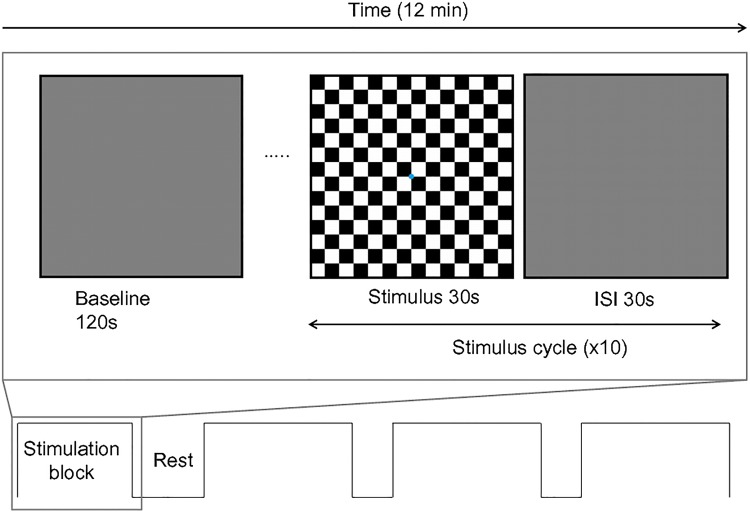
Experimental protocol showing baseline recording and cycles of visual stimulation, repeated 10x per condition.

NIRS recordings were taken from over the occipital cortices using the modified EEG 10–20 International System of Electrode Placement (Fig 1). The stimulus was a full-field reversing checkerboard with a temporal frequency of 7.5 Hz (15 reversals per second). Check sizes of 15 (small) and 30 (large) minutes of arc were used with contrasts of 50% and 100%. These parameters were used in order to be in accordance with clinical standards as outlined by the International Society for Clinical Electrophysiology in Vision[26]. It is necessary to use suprathreshold stimuli in order to characterise a reliable HDR in all adults. Participants were seated at 1 m viewing distance in a dimly lit room, testing order and sensor set-up was randomised and counterbalanced. Prior to stimulation, initial baseline measures were recorded for 120 seconds in response to a grey screen of equal mean luminance to the checkerboard. A slow event-related design was used consisting of 10 cycles of 30 seconds stimulus ‘on’ (reversing checkerboard) and 30 seconds stimulus ‘off’ (control grey screen). Blocks lasted approximately 12 minutes and participants were permitted rest breaks between recordings as required. Fig 2 shows a diagrammatic representation of the experimental procedure; note the blue fixation dot in the centre of the stimulus.

### 2.3. Data Analysis

Data pre-processing was completed with a custom-written MATLAB script. In brief, all data was normalised with respect to the pre-stimulus baseline using a simple subtraction method and a moving average filter was applied. Within-subject variance and linear drift was controlled for by this procedure. A grand average was taken of the last 15 seconds of data per epoch, representing the greatest stable change of the HDR, and differences between visual stimulation ‘on’ (reversing checkerboard) and ‘off’ (grey screen) were compared. Therefore there were 8 values per participant per chromophore (2 stimulus check sizes at both 50% and 100% contrast, recorded from O1 and O2). The normalisation procedure ensured all parametric assumptions were met for inferential statistics. Effect sizes reported are Eta-squared (η^2^) or Pearson’s correlation (r).

## Results

### 3.1. Visual HDR

**Fig 3:**
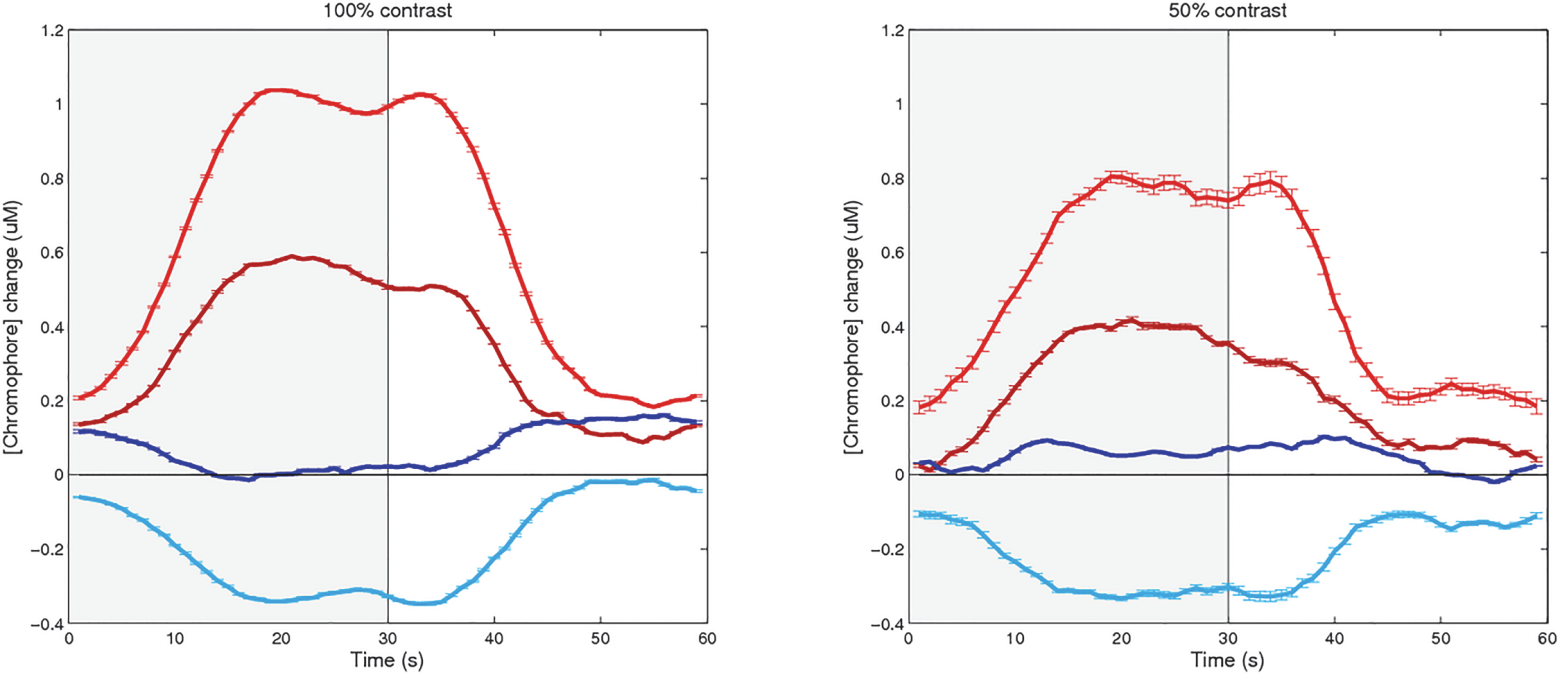
Average response cycle to visual stimulation (grey) comparing younger adults (bright) and older adults’ (dark) group HDR of an increase in [HbO] (red) and decrease in [HbR] (blue). Check sizes are averaged for each 100% contrast stimuli (left) and 50% (right), means are plotted with standard error, error bars.

Within group analyses were first conducted using a 2 x 2 x 2 repeated measures ANOVA with visual stimulus (on, off), hemisphere (O1, O2) and chromophore concentration ([HbO], [HbR]) as within subjects factors, for each stimulus manipulation (check size, contrast). As expected, both groups showed a consistent significant main effect of visual stimulation for every investigation (p<0.05), and no effect of hemisphere. During checkerboard stimulation, regardless of stimulus contrast or check size, both groups demonstrated an increase in [HbO] and decrease in [HbR] (Fig 3) as previously reported (e.g. Wijeakumar et al., 2012). However, unlike the younger group, older adults failed to present with differences between the chromophores, indicating a comparable measure of [HbO] and [HbR]. This was true for both stimulus contrasts: 100% using smaller checks (F_1, 12_ = 1.31, p>0.05) and larger checks (F_1, 12_ = 0.28, p>0.05), as well as 50% contrast using smaller checks (F_1, 9_ = 0.23, p>0.05) and larger checks (F_1, 9_ = 0.40, p>0.05). As no hemispheric differences were present, individuals’ results were averaged across the cortices generating a V1 response. Table 1 contains the descriptive statistics for the HDR to the two different stimulus check sizes at each contrast during visual stimulation.

**Table 1:** Occipital haemodynamic responses for younger (n = 12) and older (n = 13) adults at both stimulus check sizes and contrasts used (means and standard deviation reported for normalised μM data on [chromophore] change).

		100%		50%	
		Small checks	Large checks	Small checks	Large checks
[HbO]	Young	0.994 (0.38)	0.942 (0.44)	0.964 (0.45)	0.649 (0.71)
	Old	0.559 (0.56)	0.390 (0.56)	0.280 (0.42)	0.299 (0.37)
[HbR]	Young	-0.296 (0.28)	-0.306 (0.38)	-0.402 (0.44)	-0.206 (0.38)
	Old	0.032 (0.27)	0.025 (0.21)	0.078 (0.18)	0.064 (0.27)

To examine the effects of stimulus characteristics on the HDR, for each group a 2 x 2 x 2 x 2 repeated measures ANOVA was completed with stimulation (on, off), chromophore (HbO, HbR), contrast (100%, 50%) and check size (small, large) as within subjects factors. Neither group demonstrated any significant effect of either contrast or check size (p>0.05). However, there was a three-way interaction between stimulation, chromophore and contrast for each group: younger adults (F_1, 9_ = 6.93, p<0.05, μ^2^ = 0.88) and older adults (F_1, 9_ = 10.50, p<0.01, μ^2^ = 0.91). Due to the above results, post-hoc analyses were run using the ‘on’ phase only and a trend identified for [HbO] solely. In each group, pairwise comparisons revealed a trend of greater [HbO] during 100% contrast stimuli compared to 50% contrast: means of the young group were 0.97 vs. 0.80, and means of the old group were 0.55 and 0.23 respectively. Accordingly, contrast data were kept separate for all further analysis. Neither group demonstrated any effect of check size, consequently, responses to small and large check sizes were averaged together.

### 3.2. Effect of Age

**Fig 4:**
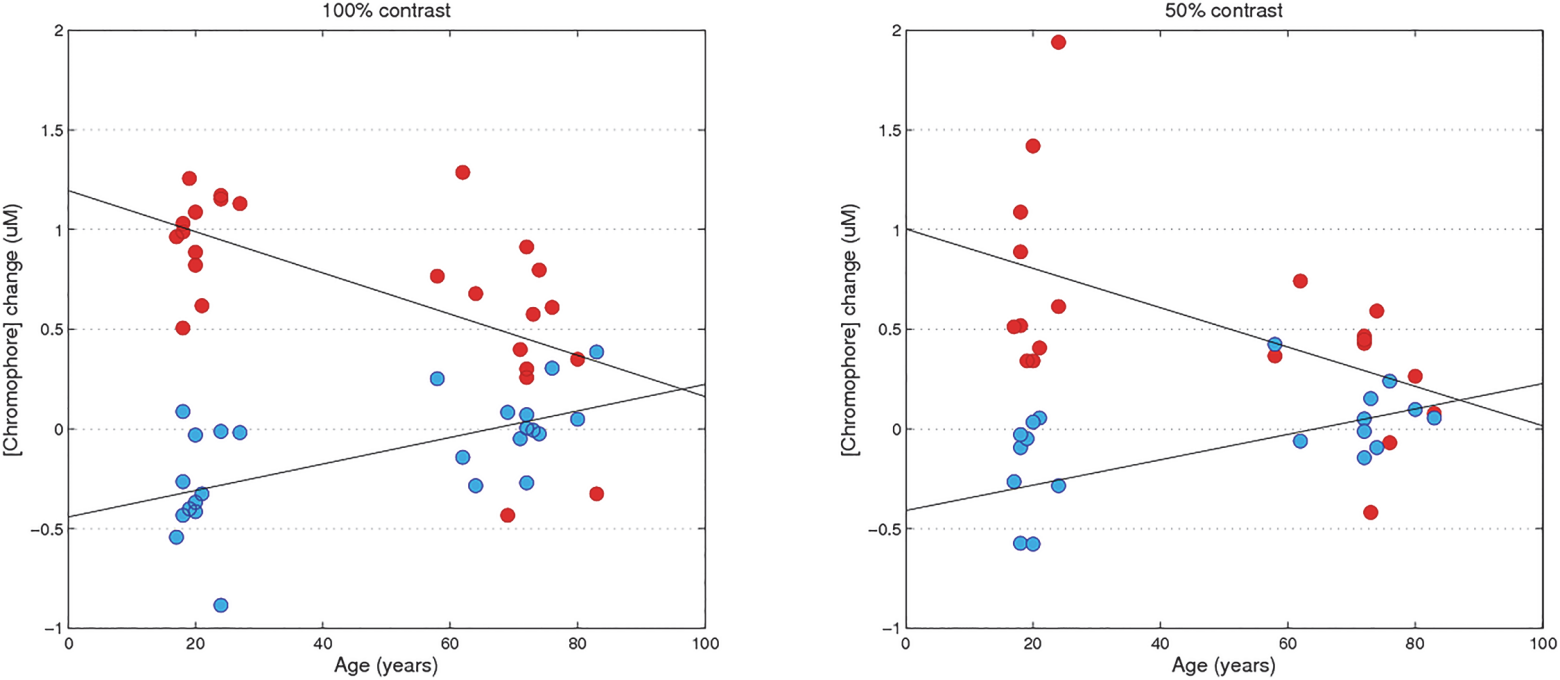
Individual grand average haemodynamic response to reversing checkerboards (average check size used). [HbO] is plotted in red, with correlation for 100% at r = -0.55, 50% at r = -0.53. [HbR] is plotted in blue, with correlation for 100% at r = 0.59, 50% at r = 0.49, all correlations are significant at p<0.01.

Group comparisons revealed a consistent effect of younger adults having significantly greater concentrations of both [HbO] and [HbR] compared to older adults. Using 100% contrast, this age-related difference was evident in both chromophores: HbO (F_1, 24_ = 10.74, p<0.01, μ^2^ = 0.91), and HbR (F_1, 24_ = 11.79, p<0.01, μ^2^ = 0.92). Similar results were found at 50% contrast: HbO (F_1, 18_ = 5.41, p<0.05, μ^2^ = 0.84), and HbR (F_1, 18_ = 8.0, p<0.05, μ^2^ = 0.89). Effect sizes were strong and overall, the variance associated with group differences, and therefore age, was substantial. Eta squared as a percentage overall for [HbO] = 88% and [HbR] = 91%, i.e. regardless of stimulus parameters, the HDR is characterised by age. Fig 4 demonstrates this differential effect of ageing on each chromophore, wherein [HbO] decreases over age and [HbR] increases. Correlations confirmed this age-related effect with [HbO] at 100% r = -0.55, at 50% r = -0.53, and [HbR] at 100% r = 0.59, 50% r = 0.49 with p<0.01 for each correlation. Due to the imbalance of sexes within our groups, we performed the same group comparison using only the females (older adults, n = 8) and found identical results: at 100%, HbO (F_1, 19_ = 17.4, p<0.01, μ^2^ = 0.95), and HbR (F_1, 19_ = 11.44, p<0.01, μ^2^ = 0.92), at 50% HbO (F_1, 13_ = 7.28, p<0.05, μ^2^ = 0.88), and HbR (F_1, 13_ = 7.27, p<0.05, μ^2^ = 0.88). By employing 50% contrast we aimed to explore whether a reduced contrast in younger adults would equate to 100% contrast responses in older adults, thereby attempting to control for the different contrast thresholds of each group. When comparing these conditions there was a similar [HbO] response for each age group (p>0.05). However, [HbR] values were significantly different with younger adults displaying greater [HbR] regardless of the reduced contrast (t_1,20_ = -2.73, p<0.05), with a mean difference of -0.36.

### 3.3. Resting HDR

**Fig 5:**
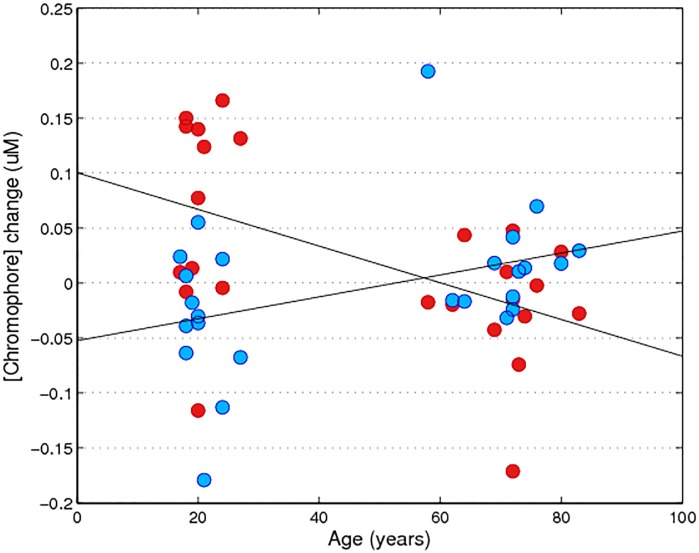
Individual grand average haemodynamic response at rest (grey screen with mean luminance to checkerboard stimulus). [HbO] is plotted in red, with correlation at R = -0.517. [HbR] is plotted in blue, with correlation at r = 0.391, p<0.05.

Resting recordings were taken prior to every testing session in response to a grey screen of equal mean luminance to the checkerboard stimulus. The task-related reduction in HDR in older adults was also present in the baseline data, indicating an age-related attenuation of [HbO] and [HbR] at rest. All baseline measures were averaged per individual and older adults showed a reduced response compared to younger adults (Fig 5), mean [HbO] 0.07 vs. -0.02 and [HbR] -0.04 vs. 0.02. This age-related effect was statistically different for both chromophores: HbO (F_1, 24_ = 9.27, p<0.01, μ^2^ = 0.29) and HbR (F_1, 24_ = 5.79, p<0.05, μ^2^ = 0.20).

**Fig 6:**
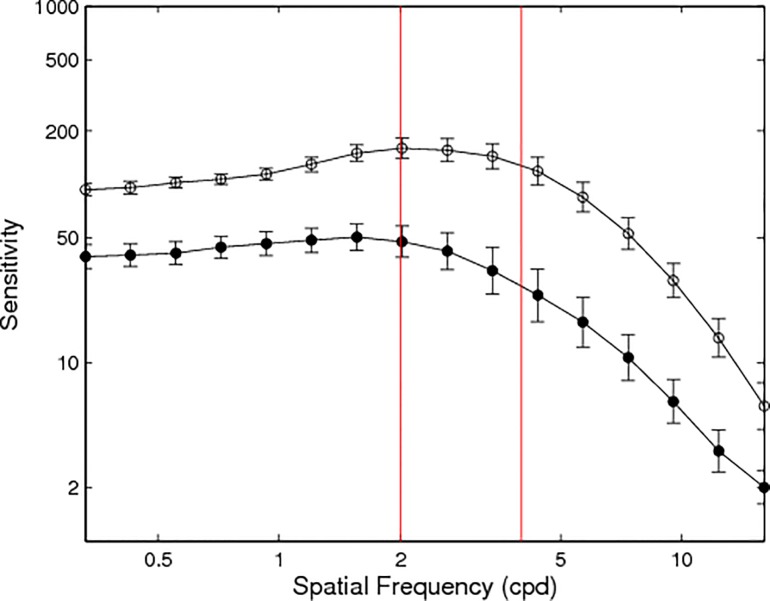
Mean quick Contrast Sensitivity Function (qCSF) per age group with young: n = 12, mean age 21, and old: n = 10, mean age 72. Black markers represent the old group average, white indicate young. Red lines demonstrate the sine-wave spatial frequencies that are analogous to our square wave checkerboard spatial frequencies.

To further investigate the impact of age on the data, correlations were run with [HbO] and [HbR], VA, visual field pattern deviation of each eye (from the Humphrey’s fields test), contrast sensitivity (qCSF) and age. Age was correlated with each chromophore with the same trend noted above: [HbO] at 100% r = -0.55, at 50% r = -0.53, and [HbR] at 100% r = 0.59, 50% r = 0.49, all p<0.01. These effects can be seen in Fig 4 with age differentially affecting chromophores. No significant relationship was identified between the optical measures (visual fields left and right eye, VA) and the fNIRS responses. As expected there was a decreased CSF associated with ageing, r = -0.706 (p<0.01) (Fig 6). A 1-way ANOVA resulted in significant group differences for the qCSF (F_1, 16_ = 15.2, p<0.01, μ^2^ = 0.94). The younger adults' group mean was 3.27, and older adults' 2.32, demonstrating reduced contrast sensitivity in older adults.

## Discussion

The intent of this study was to delineate the differences in basic visual haemodynamic response (HDR) between age groups using fNIRS. Supporting current literature [11,13,14,18,19,27,28], our results showed that both groups had characteristic responses to visual stimulation, with an increase in concentration of [HbO] and decrease in [HbR] during checkerboard presentation. These group responses were consistent regardless of stimulus parameters (checkerboard check size, contrast), demonstrating that the HDR is stable irrespective of visual stimulus characteristics.

In line with our hypothesis and previous work, older adults had a high variability of HDR, and often comparable measures of [HbO] and [HbR][20]. Fabiani et al., (2014) use their BOLD response data to conclude that this relationship between [HbO] and [HbR] may be due to a reduced MRI signal in older adults. Due to the lack of differences between HbO and HbR when recording changes in chromophore concentration, we can strongly support this hypothesis of an attenuated response. However, where Fabiani’s group failed to find any effect of visual stimulation with old adults, we found reliable differences between the grey screen and the reversing checkerboard (see Section 3.1). Perhaps our sample selection of optically healthy individuals, and increased points of data collected (40 compared to 15 blocks) can explain these differences in [HbO].

The crux of this research lies in the substantial differences between the two age groups: evidence in all comparisons of chromophores produced strong effect sizes. Our younger adults (mean age 21) consistently displayed greater concentrations of both chromophores compared to older adults (mean age 71). The effect of age accounted for 88% of the variance for [HbO] and 91% for [HbR], indicating that independent of the current sample, there is a strong age-related effect on the HDR. This is an important finding to highlight, as these measures of [HbO] and [HbR] were in response to passive visual stimulation, with all adults being healthy and fully optically corrected. No sex differences were expected within V1[29], nonetheless, when repeating the analysis with females only, the same age-related reduction in HDR was found indicating that this effect is a robust result. To our knowledge, this is the first time the age-related effects of the HDR have been examined and recorded using an FD-MD system of fNIRS, which allows for absolute [chromophore] to be measured. This study provides compelling physiological evidence of a reduced cortical response in elderly adults, regardless of conventional measures such as visual acuity, contrast sensitivity or visual fields. This directly contradicts other fNIRS findings assessing cerebral auto regulation and cerebrovascular CO_2_ reactivity in adults aged between 21–86 years old[30]. Oudegeest-Sander et al., (2014) used fNIRS, TCD, ECG and blood pressure recordings to measure oxygenation and CBF velocity in 58 adults. Although the authors conclude that ageing does not compromise CBF (TCD over middle cerebral artery) and cortical oxygenation (fNIRS over frontal cortex)[30], our results may differ simply due to the region of interest in the brain and known anterior-posterior decline of the ageing brain[31]. However, another TCD study recording from the posterior cerebral artery also failed to find any significant differences between age groups during a reading task with 60 participants aged between 10–60[32]. As we recorded fNIRS from the occipital cortex, our results are specific to the primary visual cortex and relate to a more cortical focal region of interest compared to the above studies.

Beyond task activation, we show evidence of attenuation of the older adults’ HDR during baseline measurement, i.e. when participants were viewing a grey screen at rest. In accordance with this, reduced concentrations of both HbO and HbR from the frontal cortex have been found with older and younger adults (24–91 years old) when sat upright[33], as well as in middle-aged and young adults (22–59 years old) lying supine[34,35]. Although previously an age-related reduction in CBF at rest has been reported using techniques such as TCD and MRI[8–10,32,34], this is the first study to report an occipital lobe reduction at rest in older adults recording absolute values of [HbO] and [HbR].

Many previous studies have reported stimulus characteristics, such as spatial frequency and contrast, to affect cortical responses[36–42]. For example, in fMRI, higher contrast visual stimuli produced greater response amplitudes in V1[38]. Additionally, V1 activity was consistent with perceptual responses during the psychophysical testing of contrast discrimination[38]. Zaletel et al., (2005) used both visually evoked cerebral blood flow velocity responses (VEFRs) and visually evoked potentials (VEPs) to examine reversing checkerboard responses to 3 different contrasts (100%, 10% and 1%)[37]. A linear relationship was found with higher contrasts producing increasing VEPs and VEFRS in both age groups[37]. In concurrence with Fabiani et al., (2014), Zaletel and colleagues found no age-related differences regarding the electrophysiology, but reported old adults to have a substantially reduced vascular response, similar to our findings. Interestingly, when we controlled for the different contrast thresholds and compared younger adults’ responses at 50% to those of older adults at 100%, there were similar [HbO] responses between the two age groups. This suggests that both age and contrast affects V1 [HbO] in a linear manner. However, the opposite pattern of results was found for [HbR] with younger adults consistently having greater [HbR] compared to older adults regardless of contrast. These results suggest a reduced coupling between [HbO] and [HbR] in older adults, a hypothesis supported by older adults comparable levels of [HbO] and [HbR]. Both spatial and temporal frequency has been shown to modulate the visual cortical response using fMRI[43]. VEP work has emphasised smaller checks (20 minutes of arc) to produce larger VEPs, regardless of contrast (20% and 85%)[39]. Yet, when testing young adults’ VEPs based on visual acuities, Chen et al., (2012) recommends using a larger check size (30 minutes of arc). This is in accordance with where the maximum P100 amplitude occurred for individuals with poor visual acuity[40]. Mirzajani et al., (2007) relate spatial and temporal frequencies and report that the BOLD response is maximum with a low spatial frequency (0.4 cpd) when employing a 8Hz temporal frequency[41]. Therefore by using the ISCEV clinical standard of 15 minutes of arc (4 cpd) reversing at 7.5 Hz, a tenfold difference used in the Mirzajani study, our instrumentation and design were perhaps not sensitive enough to detect the specific differences in spatial frequencies. Additionally, the quick-CSF results demonstrate that old adults have a reduced CSF compared to that of the younger group, a result supported in the literature[21,42,44]. Yet, the overall shape of the CSF is similar across groups, the relative reduction factor between the age groups being 1.4. Perhaps this similarity of the group CSF and closeness of the check sizes contributed to the lack of effect of stimulus parameters on either group’s HDR. It is conceivable that, when studying ageing with fNIRS, a greater difference between stimulus parameters or range of stimuli, similar to Boynton et al., (1999) and Zaletel et al., (2004) may produce a significantly different HDR.

A reduced HDR in ageing is reported with complex visual stimulus in much fMRI work detailing ventral stream dedifferentiation[45], face processing[46], and working memory[47]. Similarly, fNIRS over the prefrontal cortex has previously been used to quantify a reduction in [HbO] in elderly adults during verbal working memory[48], executive function[49,50] and simple mathematics[51]. Indeed, Mehagnoul-schipper et al., (2002) performed simultaneous measurements of fNIRs and fMRI during a finger tapping task and reported an age-related reduction in [HbO] and a reduced cortical area of activation[52]. Therefore, much neuroimaging work indicates that there is a reduced HDR in the ageing brain, although differences may be found between regions of interest, sample selection, task and analysis. For example, although Brodtmann et al., (2003) reported a clear distinction between young and old ventral stream activation, the authors found no systematic difference in amplitude or latency of the BOLD response across 18 elderly participants who were separated into the 7^th^, 8^th^, and 9^th^ decades[46]. Taken together, age-related decline of HDR at higher processing levels mirrors the current understanding of low level processing. While we have clearly demonstrated this attenuation in older adults’ visual cortex, the underlying processes associated with these differences are unclear. Additionally, the precise nature of these changes regarding the later quartile of life is yet unknown. Although the current sample of healthy older adults were without any neurological, ocular or vascular pathologies, our group was still very heterogeneous spanning a range of 60–81 years old. Without structural MRI scans, we cannot know the overall physiological atrophy associated with each participant which may influence chromophore concentrations as measured by fNIRS. Future work should concentrate on investigating responses from adults grouped in shorter age ranges (example 55–60, 60–65 etc.), as well as across middle and younger ages. Additionally, if future studies did recruit a range of ages across the lifespan, linear regression analysis could be used to determine the exact relationship between age and chromophore concentration. Another limitation that necessitates discussion is the choice of stimulus parameters. The current study selected fixed parameters of check size and contrast for the checkerboard to enhance group comparisons. However, future research could consider recording HDR using a wider range of stimulus parameters that would relate more closely to responses obtained both above and below individuals’ contrast detection threshold based on prior visual psychophysical testing. Subsequent work from our lab will aim to determine the specific influence of vasculature capacity and cerebral blood flow dynamics that inherently influence the HDR. By combining fNIRS with other neuroimaging and physiological techniques we hope to elaborate on if this reduction in visual HDR is due to a reduction in neuronal activation or diminished vascular capacity.

## Conclusion

To summarise, in the present study, we aimed to evaluate the impact of healthy ageing on visual cortical activity by measuring absolute changes in chromophore concentrations using fNIRS. Our data show that older adults had significantly reduced [HbO] and [HbR] compared to young adults, regardless of visual acuity, contrast sensitivity or ocular health. Effect sizes were remarkably strong – the average variance associated with age for [HbO] was 88% and HbR 91%. These results indicate that in passive viewing of basic checkerboard stimulation, without any cognitive input, there is an age-related decline in HDR. Moreover, regardless of stimulus parameters such as contrast and check size, the HDR is characterised by age. In concurrence with present neuroimaging literature, we conclude that the visual HDR decreases as healthy ageing proceeds.
